# Optimizing Oncolytic Viral Design to Enhance Antitumor Efficacy: Progress and Challenges

**DOI:** 10.3390/cancers12061699

**Published:** 2020-06-26

**Authors:** Shyambabu Chaurasiya, Yuman Fong, Susanne G. Warner

**Affiliations:** Department of Surgery, City of Hope National Medical Center, Duarte, CA 91010, USA; schaurasiya@coh.org (S.C.); yfong@coh.org (Y.F.)

**Keywords:** oncolytic virus, transgene, arming, immunotherapy, Cytokines, CAR-T cells

## Abstract

The field of oncolytic virotherapy has seen remarkable advancements in last two decades, leading to approval of the first oncolytic immuno-virotherapy, Talimogene Laherparepvec, for the treatment of melanoma. A plethora of preclinical and clinical studies have demonstrated excellent safety profiles of other oncolytic viruses. While oncolytic viruses show clinical promise in already immunogenic malignancies, response rates are inconsistent. Response rates are even less consistent in immunosuppressed tumor microenvironments like those found in liver, pancreas, and MSI-stable colon cancers. Therefore, the efficacy of oncolytic viruses needs to be improved for more oncolytic viruses to enter mainstream cancer therapy. One approach to increase the therapeutic efficacy of oncolytic viruses is to use them as primers for other immunotherapeutics. The amenability of oncolytic viruses to transgene-arming provides an immense opportunity for investigators to explore different ways of improving the outcome of oncolytic therapy. In this regard, genes encoding immunomodulatory proteins are the most commonly studied genes for arming oncolytic viruses. Other transgenes used to arm oncolytic viruses include those with the potential to favorably modulate tumor stroma, making it possible to image the virus distribution and increase its suitability for combination with other therapeutics. This review will detail the progress made in arming oncolytic viruses with a focus on immune-modulatory transgenes, and will discuss the challenges that need to be addressed for more armed oncolytic viruses to find widespread clinical use.

## 1. Introduction

Oncolytic viruses (OVs) represent a novel class of therapeutics that use replication-competent, live viruses to treat cancer. OVs are either inherently cancer-selective or are genetically modified to replicate specifically in cancer cells. Cancer cells accrue myriad mutations during the course of evolution which allow them to evade the immune system and grow in an unchecked manner [1]. Interestingly, while preparing themselves for immune evasion, cancer cells often inactivate their antiviral protection mechanisms, such as the interferon pathways, leaving themselves vulnerable to viruses [2]. First-generation OVs utilize these mutated loopholes to specifically replicate in and kill cancer cells while leaving normal cells unharmed. Examples of first-generation OVs include reovirus [3], vesicular stomatitis virus (VSV) [4] and Newcastle disease virus (NDV) [5]. Unlike first-generation OVs which are inherently cancer selective, second-generation OVs are genetically manipulated to selectively replicate in cancer cells. A wide variety of viruses including adenovirus [6], herpes simplex virus (HSV) [7], vaccinia virus (VACV) [8], poliovirus [7], myxomavirus [9] and measles virus [10] have been genetically altered to make them cancer selective. Furthermore, these second-generation oncolytic viruses have also been armed with transgenes to activate antitumor immunity or to aid to the efficacy of OVs in some other way. These armed viruses are often referred to as third generation OVs. This review will discuss armed viruses with a primary focus on immunomodulatory transgenes.

## 2. Oncolytic Virus and the Immune System

Until one decade ago, the immune system was largely assumed to be one of the biggest hurdles in the success of oncolytic virus. This assumption was based on the premise that antiviral immune response not only causes premature clearance of the virus, but also hampers subsequent rounds of treatment with the virus [11,12,13]. However, the field has evolved to view the immune system not as a hurdle, but as an essential component for the success of OV therapy [14,15,16,17,18,19]. It should be noted that OVs induce not only antiviral immunity, but also antitumor immunity. While antitumor immunity is obviously beneficial in the treatment of cancer, antiviral immunity does not necessarily contravene the efficacy of OVs. In fact, antiviral immunity is thought to be essential for the initial priming of antitumor immunity, as well as for the conversion of immunologically “cold” tumors into immunologically “hot” ones (reviewed by Gujar et al. [19]). Immunologically “cold” tumors display one or more of the following features: low levels of tumor antigen, lack of T cells recognizing tumor antigens, low levels of CD8+ T cells, high levels of immune suppressive cells and/or cytokines. In contrast, immunologically “hot” tumors have high levels of tumor antigens, high levels of effector T cells and low levels of immune suppressive cells and/or cytokines. Furthermore, since OVs preferentially infect cancer cells, immune cells directed against virus antigens will also kill cancer cells [19].

OVs kill cancer cells mostly by inducing immunogenic cell death (ICD) [20,21,22]. This type of cell death, which includes immunogenic apoptosis, pyroptosis, necrosis, necroptosis and autophagy, causes the release of a plethora of damage-associated molecular patters (DAMPs) such as ecto-calreticulin, ATP, heat shock proteins, HMGB1, uric acid as well as pathogen-associated molecular patterns (PAMPs) [20,21]. In addition to DAMPs and PAMPs, a repertoire of tumor-specific antigens (TSAs) and/or tumor-associated antigens (TAAs) is also released from the dying cancer cells [20]. Danger signals provided by DAMPs and PAMPs, together with the TSA/TAA, are required for dendritic cells to evoke an adaptive antitumor immunity [23,24]. Given their role in the activation of antitumor immunity, OVs are now considered a type of immunotherapy [25].

## 3. Strategies for Arming Oncolytic Viruses

Cancer is a complex disease, and equally complex treatments will be needed to eliminate it. In complex solid tumors, it is almost impossible to infect all cancer cells, no matter how enormous the dose of the virus administered and what route is used to administer the virus. Compared to intratumoral injection, OVs administered systemically are more prone to be cleared by different components of the host immune system, including complement and neutralizing antibodies. Hence, systemically delivered OVs are less efficient at reaching the tumor sites. Investigators have studied different strategies to protect OVs from immune components, including the use of stem cells, immune cells and cancer cells as carriers to deliver OVs. Furthermore, bio-compatible polymers such as polyethylene glycol have also been used to coat OVs in order to protect them from host immune components. While OVs, in theory, can self-amplify within the tumor and possibly infect all cancer cells, this scenario will be possible only if there is nothing to obstruct the replication and spread of the virus. However, in real-life, that is not the case. Clinical trials with OVs have revealed that virus titer rapidly declines within days after injection [26,27]. Therefore, it is highly unlikely that OVs could infect and kill all cancer cells in a tumor. Furthermore, while OVs possess the inherent ability to induce antitumor immunity, the extent of the antitumor activation may not be optimal for maximal tumor destruction. Hence, it is desirable to arm OVs with transgenes coding for proteins that can directly or indirectly aid overall antitumor efficacy. The ability of OVs to specifically infect cancer cells makes them good vectors for the localized expression of transgenes within the tumor microenvironment (TME), thereby reducing the potential toxicities associated with the systemic delivery of the products of the transgenes. Furthermore, the temporal regulation of gene expression in viruses, as well as existence of promoters with different transcriptional strengths, provides flexibility in controlling the timing and extent of transgene expression from oncolytic viruses. For example, gene expression in the poxvirus is programmed into three temporal stages: early, intermediate and late [28,29]. While early and intermediate genes are expressed before DNA replication, late gene expression is initiated only after the replication of the viral genome [28,29]. Therefore, a transgene placed under the control of a late promoter will only be expressed in cells that support replication of the virus. Furthermore, investigators have made synthetic promoters which can further regulate the expression of transgenes [30].

A variety of genes with a wide range of functions including immune-stimulation and stromal-modulation, have been used to arm different types of oncolytic viruses. Some of the most commonly studied oncolytic viruses include adenovirus, herpes simplex virus, poxvirus and reovirus. Table 1 compares and contrasts the properties of these four viruses. While many of the armed viruses are still in the preclinical phase, more than a dozen of them are currently in clinical trials (Table 2); the only OV to have received FDA approval, Talimogene laherparepvec (T-VEC), is also an armed OV.

## 4. Oncolytic Viruses Armed to Modulate Antitumor Immunity

Generating an adaptive antitumor immune response is a complex, multistep process, starting with the uptake of tumor antigens by antigen presenting cells and ending with the activation of effector T cells that can recognize and kill the tumor cells [34]. However, tumors employ strategies to thwart antitumor immune response by interfering at almost every step of immune activation [35]. Tumor cells, with the help of stromal cells, maintain an immune-suppressive environment within TME [35,36]. While OVs could inherently revert the immune-suppressed tumor microenvironment to some extent [19,37], different immune-modulating genes have been used to arm OVs to further increase the antitumor immune activating property of OVs and ultimately increase antitumor efficacy and reduce the probability of disease relapse. Genes encoding cytokines, chemokines, inhibitors of immune checkpoints, bi-specific T cell engagers, tumor antigens, and targets for chimeric antigen receptor (CAR)-T cells have been used to arm OVs with the purpose of enhancing antitumor immunity.

## 5. Cytokines

**Figure 1:**
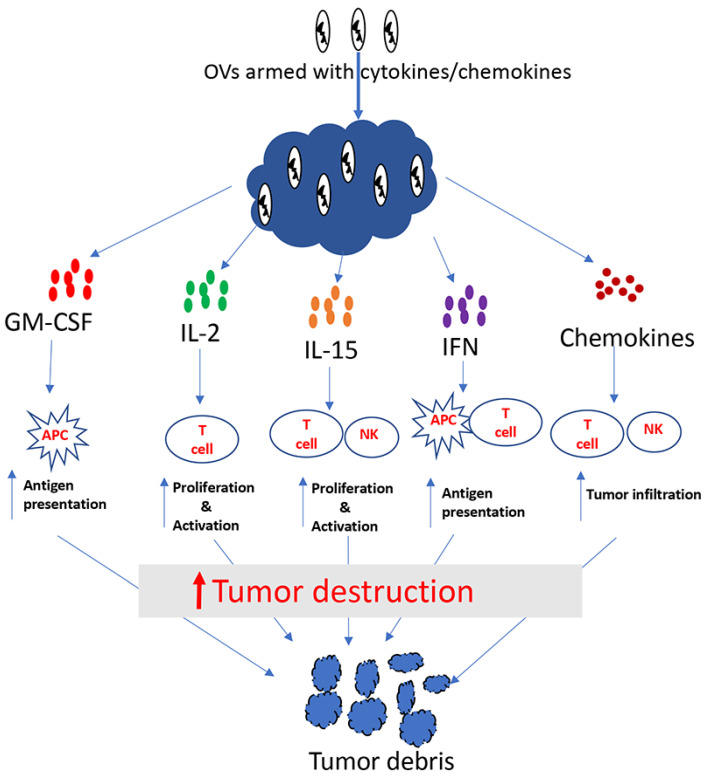
Tumor destruction by cytokine/chemokine-armed oncolytic viruses.

Cytokines are soluble proteins that allow immune cells to communicate. They play an important role in the regulation of both the innate and adaptive immune system. Some crucial aspects of the immune system, including differentiation, proliferation, effector functions and survival of immune cells, are controlled by cytokines [38,39]. Many cytokines including granulocyte macrophage colony stimulating factor (GM-CSF), interleukin (IL)-2, IL-12, IL-15, interferon (IFN)-α, IFN-β and IFN-γ have shown antitumor properties in preclinical studies (Figure 1). Among these cytokines, IFN-α [40] and IL-2 [41] were approved by FDA more than two decades ago for the treatment of some malignancies. Cytokines generally have short half-lives and act over short distances, which is why high doses are used for systemic administration, so that an effective dose can reach the tumor [39,40,42]. Unfortunately, high doses of cytokines often result in severe toxicities. For example, high doses of IL-2 can cause life-threatening side-effects such as vascular leak syndrome [43,44]. Hence, it is logical to surmise that localized expression of cytokines within TME will not only increase their antitumor efficacy, but also reduce systemic toxicity. With this idea, many investigators have used cytokine-encoding genes to arm oncolytic viruses.

### 5.1. Granulocyte Macrophage Colony Stimulating Factor (GM-CSF)

GM-CSF is one of the most commonly used cytokines for arming oncolytic viruses. GM-CSF is produced from activated T cells, fibroblasts, endothelial cells, macrophages and stromal cells [45]. GM-CSF helps in antigen presentation through recruitment and activation of dendritic cells and macrophages [46]. Several types of viruses, including HSV, VACV and adenovirus, have been armed with GM-CSF and evaluated in preclinical as well as clinical studies in different types of cancer. T-VEC, the only OV that has received FDA approval so far, is armed with GM-CSF. T-VEC is an HSV-1 (JS-1 strain) with both copies of the neurovirulence gene *ICP34.5* replaced with two copies of human GM-CSF cDNA [47]. Also, T-VEC is deleted in the *ICP47* gene, whose product inhibits the transporter associated with antigen presentation (TAP). The deletion of *ICP34.5* genes increases the safety of T-VEC, while the deletion of *ICP47* allows for MHC-I-dependent antigen presentation from virus-infected cells; the virus-encoded GM-CSF helps in the recruitment of DC, and promotes their antigen presenting function [48]. Another clinically advanced OV, called Pexa-Vec, is also armed with GM-CSF. Pexa-Vec is a Wyeth strain of the vaccinia virus with the *J2R* gene deleted, encoding for viral thymidine kinase, and a cDNA encoding human GM-CSF inserted at the *J2R* locus [49]. Pexa-Vec has completed multiple phase I and II trials with some degree of success in patients with different types of malignancies [50,51]. Likewise, adenoviruses armed with GM-CSF have also been studied in phase I clinical trials and found to be safe with modest antitumor efficacy [52,53].

### 5.2. Interleukin-2

IL-2 is primarily secreted from CD4+ T cells, but it can also be secreted from CD8+ T cells, NK cells and dendritic cells [54,55]. This cytokine potently induces the activation and proliferation of T cells. In 1992, IL-2 became the first cancer immunotherapy to be approved by the FDA for the treatment of metastatic renal cancer, and in 1998, its use was approved for the treatment of metastatic melanoma [41]. Because high levels of IL-2 delivered systemically can result in severe toxicity, investigators have tried to locally express IL-2 in tumors using different viral vectors [44,56]. Likewise, OVs have also been armed with IL-2. In a recent study, Liu et al. used the IL-2 gene to arm an oncolytic vaccinia virus and studied safety as well as antitumor efficacy of the virus in different murine tumor models [57]. The IL-2 transgene used in this study was modified to contain a glycosylphosphatidylinositol anchor with a rigid peptide linker in order to maintain IL-2 within TME to reduce toxicity. The virus was found to be effective in treating tumors with no side effects.

### 5.3. Interleukin-12

Many preclinical studies have shown that IL-12 has potent antitumor activity (reviewed by Weiss et al. [58]). In addition to the activation of antitumor immune cells (T cell and NK cells), IL-12 has also been shown to negatively affect tumors through its antiangiogenic activity [59]. Several OVs have been armed with IL-12 and studied for the treatment of different types of cancer. For example, Hellums et al. found higher levels of CD4+, CD8+ and NK cells in syngeneic murine gliomas treated with an IL-12-armed oncolytic HSV-1 compared to tumors treated with an unarmed oncolytic HSV-1 [60]. Consequently, the IL-12-armed oncolytic HSV-1 resulted in better survival compared to the unarmed virus. Ge et al. studied the safety and antitumor efficacy of an oncolytic vaccinia virus encoding a membrane-bound version of IL-12 [61]. The virus was found to convert an immunologically “cold” tumor into a “hot” one, and to increase the survival of mice bearing syngeneic colon tumors. Furthermore, when combined with a PD-1 inhibitor, the virus resulted in complete tumor regression in all mice that had late-stage colon cancer. Other oncolytic viruses, including adenovirus [62,63], VSV [64,65] and Semliki Forest virus [66], have also been armed with IL-12, and shown better efficacy compared to their respective unarmed parental viruses.

### 5.4. Interleukin-15

The cytokine IL-15 promotes the proliferation, activation and survival of CD8+ T cells, NK cells, NKT and dendritic cells [67]. IL-15 shares two of the three subunits of the IL-2 receptor, and has some overlapping functions [67]. Despite similarities in function, several studies suggest that IL-15 is more potent in controlling tumors and is less toxic than IL-2 [68,69]. A number of oncolytic viruses have been armed with the IL-15 gene and have shown superior antitumor efficacy compared to their unarmed counterparts [70,71,72]. A recent study by Kowalsky et al. armed an oncolytic vaccinia virus with IL-15 superagonist (a fusion protein of IL-15 and IL-15Rα) and found the virus to induce strong antitumor immunity, resulting in better therapeutic benefits in murine tumor models, which were further enhanced when the virus was combined with PD-1 blockade [73].

### 5.5. Interferons

The name “interferon” was originally applied to some cytokines due to their ability to interfere with virus replication. Type I interferons, including IFN-α and IFN-β, are potent antiviral cytokines [74]; however, that has not stopped investigators from using these cytokines to arm oncolytic viruses. The rationale for using Type-I IFN as a transgene in oncolytic viruses is that, apart from their antiviral function, they also have antitumor potential due to their role in the maturation of dendritic cells [75] and cytotoxic T cells [76]. An oncolytic adenovirus armed with IFN-α and used in combination with radiation was found to yield a modest increase in antitumor efficacy in murine models of pancreatic cancer [77]. Likewise, some degree of improvement in antitumor efficacy of oncolytic VACV [78], VSV [79], NDV [80] and measles [81] were observed after arming them with IFN-β.

IFN-γ, the only type-II IFN, has also been shown to exert an antitumor effect through a variety of mechanisms [82,83]. First, IFN-γ can inhibit the proliferation of cancer cells and also induce apoptosis [84]. Second, it affects tumors by inhibiting angiogenesis [85]. Lastly, it induces innate and adaptive immunity against cancer [86]. Commensurate with the antitumor role of IFN-γ, an oncolytic VSV engineered to encode IFN-γ exerted superior antitumor effect in multiple types of murine tumor models [87]. Compared to the parental virus, the IFN-γ-encoding virus was found to be more potent in the activation of dendritic cells, and it also resulted in higher levels of proinflammatory cytokines in the treated mice.

## 6. Chemokines

These are small secreted proteins that act as chemoattractant for leukocytes [88] (Figure 1). Because of their ability to attract immune cells, it may be beneficial to arm OVs with chemokines, especially for the treatment of immunologically “cold” tumors that lack effector immune cells. Indeed, several OVs have been armed with chemokines, resulting in enhancements of the antitumor efficacy. Nishio et al. armed an oncolytic adenovirus with the chemokine RANTES (CCL5) and the cytokine IL-15. They studied the antitumor potency of this virus in combination with CAR-T cells. The authors found that RANTES and IL-15 expressed from the oncolytic virus within the tumor was able to increase tumor infiltration by the CAR-T cells that were delivered systemically [88]. Likewise, Liu et al. used the chemokine CXCL11 to arm an oncolytic VACV, and studied its oncolytic potency in a mesothelioma model [89]. CXCL11 arming was found to attract higher numbers of tumor-specific CD8+ T cells compared to parental virus; consequently, higher therapeutic benefits were achieved with the CXCL11-encoding virus. Another study by Li et al. reported the enhanced safety and antitumor efficacy of an oncolytic VACV armed with CCL5 [90]. Since immunologically “cold” tumors are mostly refractory to immunotherapy, increasing effector immune cells in the tumor through the expression of chemoattractants from OVs seems to be an attractive approach for cancer therapy.

## 7. Inhibitors of Immunosuppressors

Tumors maintain an immune-tolerant microenvironment through the activation of a plethora of immune-suppressive mechanisms [35]. The immune-suppressed environment within tumors not only inhibits immune cells from mounting antitumor effects, but also reduces the efficacy of immunotherapy. A variety of cellular and secreted factors play crucial roles in maintaining immune suppression. Different strategies have been studied to arm OVs in order to overcome these immune-suppressive factors.

### 7.1. Inhibition of Secretory Immunosuppressive Factors

Some of the most potent secreted immune-suppressive factors are transforming growth factor-beta (TGF-β), vascular endothelial growth factor (VEGF) and prostaglandin E2 (PGE2) [91]. TGF-β inhibits the activation, proliferation and differentiation of T cells, and hence, negates their cytotoxic ability [92]. Likewise, VEGF has been shown to induce the proliferation of immunosuppressive cells, prevent T-cell recruitment to tumors, and induce exhaustion in T-cells [93]. Similarly, PGE2 exerts its immunosuppressive function through interference with antigen presentation by dendritic cells and by fostering the proliferation of myeloid-derived suppressor cells (MDSCs) [94]. Therefore, it is plausible that expression of proteins from OVs against these immunosuppressive cytokines may boost the antitumor efficacy of OVs. With this rationale, investigators have engineered OVs to encode proteins that can specifically inhibit TGF-β, VEGF or PGE2. For example, Yang et al. created an oncolytic adenovirus that encodes a soluble receptor for TGF-β, which can trap TGF-β and abrogate its immunosuppressive function [95]. The virus was found to increase Th1 cytokine, granzyme and perforin and CD8+ T cells in tumors, and promote the generation of CD4+ memory cells. Conversely, the virus reduced Th2 cytokines, the number of regulatory T (Tregs) cells and bone marrow-derived suppressor cells. Consequently, the virus demonstrated superior antitumor efficacy compared to the parental virus. Likewise, an oncolytic VACV-encoding antibody against VEGF was shown to increase innate immune cells in tumors, resulting in better therapeutic efficacy [96]. In another study, Hou et al. identified PGE2 as a key factor allowing tumors to resist immunotherapies as well as oncolytic virotherapy. In order to inhibit PGE2-mediated resistance, the authors armed an oncolytic VACV with a PGE2-inactivating enzyme, hydroxyprostaglandin dehydrogenase (HPGD) [97]. The HPGD-encoding virus was able to overcome localized immunosuppression and increase tumor infiltration by effector T cells. The armed virus exhibited better antitumor efficacy and sensitized previously resistant tumors to immune-checkpoint inhibitors.

### 7.2. Inhibition of Cellular Immunosuppressive Factors

T cells express checkpoint receptors such as CTLA-4, PD-1, LAG-3 and TIM-3. When these receptors interact with their ligands expressed on cancer cells, stromal cells or antigen presenting cells, the T cells become dysfunctional [98]. Because the checkpoint receptors need to physically interact with their ligands to inactivate T cells, it is possible to block such interactions using antibodies against the receptors or ligands [99]. Unleashing the antitumor potential of CD8+ T cells through the use of immune checkpoint inhibitors (ICI) can result in a strong antitumor effect. Indeed, ICIs have revolutionized the field of cancer immunotherapy with unprecedented, long-lasting therapeutic efficacies in many types of cancer [100]. However, only a small fraction of patients with immunologically “hot” tumors seem to benefit from ICIs [101]. OVs have been shown to convert immunologically “cold” tumors into “hot” tumors, suggesting that they could sensitize otherwise resistant tumors to ICIs [102,103]. Furthermore, we and others have shown that cancer cells upregulate the expression of checkpoint ligands in response to oncolytic viruses [103,104]. Together, these observations indicate that OVs and ICIs will make an attractive combination with which to fight cancer. Based on this idea, many studies have examined the combination of oncolytic viruses with antibodies against checkpoint ligands or receptors, and based on encouraging preclinical results, numerous clinical trials are currently underway to evaluate the combo therapy in multiple tumor types (Table 2).

At present, both T-VEC and approved ICIs cost well over one hundred thousand each for one course of treatment [105,106]. Given the high cost of ICIs, it will be beneficial to have ICIs encoded from the oncolytic virus itself. Indeed, many OVs have recently been engineered to encode ICIs. An oncolytic VACV encoding PD-1 antibody showed better antitumor efficacy compared to the parental virus, and the efficacy of the armed virus was similar that of the parental virus in combination with systemically administered PD-1 antibodies [107]. In another study, Bartee et al. engineered an oncolytic myxoma virus to encode a soluble form of PD-1 to inhibit the PD1/PD-L1 pathway [108]. Intratumoral injection of this virus in a murine melanoma model resulted in antitumor efficacy that was greater than that of the parental virus combined with the systemic injection of PD-1 antibodies. Similarly, recently, Wang et al. reported the construction and characterization of an oncolytic vaccinia virus encoding a PD-L1 inhibitor and GM-CSF. Using different tumor models, the authors showed that the expression of a PD-L1 inhibitor from the virus increased overall efficacy of the virus [109]. In another study, Dias et al. vectorized a CTLA-4 antibody in an oncolytic adenovirus and observed a modest increase in antitumor efficacy in prostate and lung cancer models [110]. In contrast to all these studies where vectorization of ICIs in OVs enhanced the antitumor efficacy of the viruses, a study by Engeland et al. found no difference between the efficacy of an oncolytic Measles virus encoding anti-PD-L1 or anti-CTLA-4 and the unarmed parental virus in a murine melanoma model [111]. However, systemically administered anti-PD-L1 or anti-CTLA-4 recombinant protein did increase the antitumor efficacy of the parental virus, suggesting that the route of administration for ICIs may determine their antitumor efficacy.

## 8. Tumor Antigens

Some types of tumors with low mutation burdens may not have adequate levels of neo-antigens; hence, it could be difficult for CD8+ T cells to recognize and eliminate those tumors. Ovarian cancer is an example of cancer with low mutation burden [112]. One way to increase the abundance of tumor antigens would be to use an OV armed with a tumor antigen. A recent study by McGray et al. demonstrated the feasibility of such an approach to boost vaccine priming and increase overall therapeutic response in an ovarian cancer model [113]. The authors used a Maraba virus encoding full-length ovalbumin (OVA) as a tumor antigen to treat a murine ovarian tumor model engineered to express OVA. Mice bearing ovarian tumors were first immunized with OVA admixed with an adjuvant, and then treated with OVA-encoding oncolytic Maraba virus. This prime-boost strategy using tumor antigen-armed OV greatly increased tumor antigen-specific CD8+ T cells, and resulted in improved therapeutic response.

## 9. Bispecific T Cells Engager (BiTE)

One approach to increasing the T cell-mediated killing of cancer cells is to use a bispecific antibody which can bind to T cells (CD3) and a target antigen on the surface of cancer cells [114]. BiTEs bind to T cells and cancer cells simultaneously and facilitate the lysis of cancer cells, even in the absence of costimulatory signals [115]. So far, one BiTE, called Blinatumomab, has been approved by the FDA for the treatment of B-cell malignancies [116], and many others are currently under clinical trials (*clinicaltrials.gov* accessed in May 2020). Many investigators have studied the potential of arming oncolytic viruses with BiTEs to achieve higher therapeutic responses. For example, Yu et al. engineered an oncolytic VACV to encode a secretory BiTE that could engage T cells with the tumor cell surface antigen EphA-2 [117]. The virus, in combination with T cells, demonstrated higher antitumor efficacy in a xenograft model of lung cancer. Likewise, Fajardo et al. constructed an oncolytic adenovirus armed with BiTE specific for EGFR and CD3. In an in vitro coculture experiment, oncolysis by BiTE-encoding OV greatly enhanced T-cell activation, proliferation and cytotoxicity [118]. Also, the BiTE-encoding adenovirus increased the persistence and accumulation of T cells in tumors and resulted in higher antitumor efficacy compared to parental virus in xenograft models. Another study using BiTE to arm an OV was recently published by Freedman et al., where they took a different approach from other investigators [119]. In this study, the BiTE was designed to target cancer-associated fibroblasts (CAFs) instead of directly targeting cancer cells. BiTE encoded from the virus was specific for fibroblast activation protein, a protein overexpressed on CAF, and CD3 on T cells. The virus was tested in malignant ascites and prostate biopsies freshly obtained from patients. As expected, BiTE-encoding OV increased T-cell mediated killing of CAFs, which led to the depletion of CAF-mediated immunosuppression, upregulation of proinflammatory cytokine and increased antigen presentation and T cell function in ascites. This study serves as a proof-of-principle that it is possible to gain immune-mediated antitumor responses simply by blocking immune-suppressive stromal cells.

## 10. Target for CAR-T Cells

**Figure 2:**
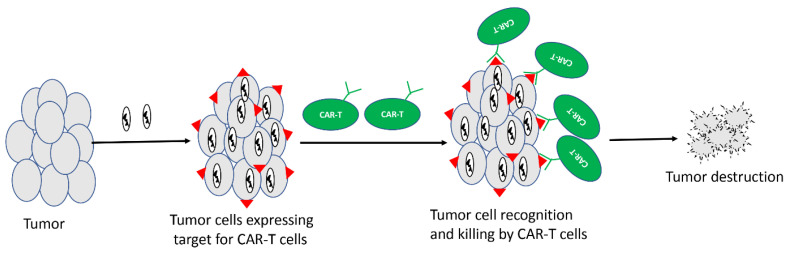
Oncolytic viruses delivering target for CAR-T cells. Unique antigens can be delivered to tumor cells using oncolytic virus, and CAR-T cells specific for that unique antigen can be used in combination to destroy tumors.

CAR-T cells are T cells redirected against tumors through engineered expression of CARs. These modified T cells are injected into cancer patients where they can specifically kill tumor cells. CAR-T cells directed against CD19 work very well against B cell malignancies, and so far, two CD-19-specific CAR-Ts, namely Kymriah [120] and Yescarta [121], have received FDA approval for the treatment of different types of B cell malignancies. However, unlike in B cell malignancies, CAR-T cells have shown suboptimal efficacy in solid tumors. One of the major reasons for the limited success of CAR-T in solid tumors is the lack of the selective and homogeneous expression of antigens on tumor cells [122]. Given the ability of OVs to selectively infect cancer cells, it may be possible to selectively express a unique antigen from cancer cells by using an antigen-armed OV, followed by treatment with CAR-T cells directed against that antigen (Figure 2). A recent study by Aalipour et al. demonstrated proof-of-principle for such a strategy [123]. In this study, the authors used an oncolytic VACV to selectively deliver CD19 to tumor cells in a murine melanoma model, and used CAR-T cells directed against CD19. The combination of CD-19-directed CAR-T with CD19-encoding OV resulted in improved survival of mice compared to antigen-mismatched combinations.

## 11. Stromal Modifiers

**Figure 3:**
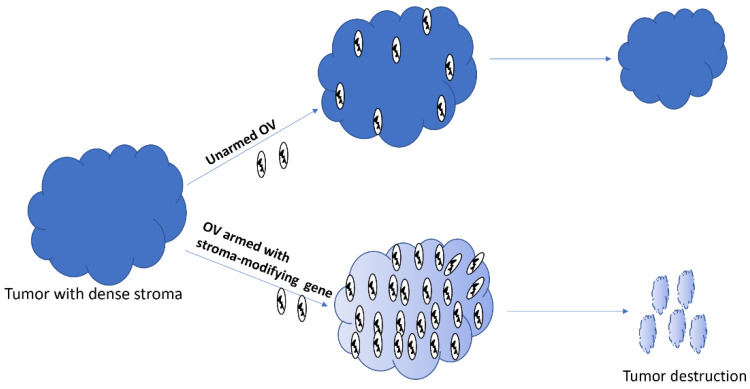
Oncolytic viruses armed with stroma-modifying genes can spread better within the tumor, and subsequently destroy it.

Oncolytic viruses administered intratumorally or through systemic routes can access tumors; however, their ability to spread within the tumors is mired by stromal components, including extracellular matrix (ECM) proteins [124,125]. Collagen and hyaluronic acid are two major components of ECM that increase interstitial fluid pressure and block therapeutics from reaching cancer cells [126]. Therefore, arming OVs with genes whose products can destroy the stromal factors could potentially increase the spread of OVs within dense solid tumors, and hence, improve the overall therapeutic efficacy (Figure 3). Indeed, an OV armed with the proteolytic enzyme hyaluronidase showed better intratumoral spread and higher antitumor efficacy in a xenograft model of melanoma in mice [127]. Likewise, an oncolytic VACV engineered to express metalloproteinase 9 was shown to degrade collagen IV and spread better in prostate cancer xenograft models. Consequently, the armed virus resulted in higher antitumor efficacy compared to the unarmed parental virus. Many other genes with the potential to modify stromal components, including decorin [128] and relaxin [129], have also been used to arm OVs.

## 12. Transgenes for Imaging

The ability to noninvasively track OVs using molecular imaging allows researchers to monitor the safety and efficacy of the virus in real time. Two types of molecular imaging exist: optical and deep-tissue. Many oncolytic viruses have been armed with transgenes to facilitate such imaging. While fluorescence- or luminescence-based optical imaging works well for preclinical studies, it may not be adequate for clinical purposes. Therefore, it is preferable to arm viruses with transgenes that will facilitate deep tissue imaging using MRI, PET or SPECT scanners (reviewed by Haddad et al. [130]). For optical imaging, transgenes encoding florescent proteins such as green fluorescent protein (GFP) and red fluorescent protein (RFP) are commonly used, whereas Firefly luciferase, Renilla luciferase and Gaussia luciferase are commonly used as transgenes for the bioluminescence-based optical imaging of viruses [130,131,132]. For deep tissue imaging, oncolytic viruses are armed with genes whose products work as prodrug converting enzymes or receptors or symporter/transporter. For example, the thymidine kinase gene from HSV-1 (HSV-tk) is used as a prodrug converting enzyme that phosphorylates the membrane permeable radiolabeled prodrug ganciclovir and traps it inside the cells, enabling PET imaging of virus-infected tissues/tumors [133]. Another approach to facilitate deep tissue imaging is the use of the human somatostatin receptor 2 (hSSR2) gene in oncolytic viruses. The expression of hSSR2 on the surface of infected cells binds to the radiolabeled, high-affinity, synthetic peptide pentetreotide and allows SPECT-based imaging to track virus infection [134,135]. The third strategy for deep tissue imaging uses a sodium iodide symporter (NIS) gene to arm oncolytic viruses. Virus-encoded NIS protein allows the uptake of radioisotopes such as ^124^I, ^131^I and rhenium by the infected cells. The NIS protein allows the uptake of most radioisotopes that have been approved for use in humans. Several studies have shown the potential of the NIS gene for imaging oncolytic viruses [136,137]. In our lab, we generated a chimeric oncolytic poxvirus and armed it with the human NIS gene under the control of a synthetic early/late promoter [136]. We used ^124^I to successfully image the virus infection and spread in murine models, and ^131^I to increase the therapeutic efficacy of the virus.

## 13. Discussion and Future Directions

Oncolytic viruses are multi-mechanistic bio-therapeutics against cancer. In addition to the direct lysis of cancer cells, OVs also activate antitumor immunity and damage tumor vasculature [138]. Most OVs are amenable to the incorporation of transgenes in their genome. This aspect confers upon OVs an unlimited possibility for modifications with the goal of increasing safety and antitumor efficacy. It is possible to modify OVs to encode one or more transgenes, each of which can be a therapeutic on its own. Not surprisingly, a wide range of therapeutic genes have been used to arm OVs. Most of the transgenes used to arm OVs possess immune-stimulatory function. One of the reasons why immunotherapy is so appealing is that antitumor response to immunotherapy can be long-lasting, due to the generation and maintenance of tumor-specific memory T cells [101]. Furthermore, the use of OVs to express immune-modulatory therapeutics has several advantages over the combination of OV with exogenous immune-therapeutics. First, immune-therapeutics such as cytokines have short half-lives and act over short distances, which is why repeated injections of high doses are required to achieve meaningful antitumor effects. However, OV-encoded cytokines can be restricted to TME and, at least in theory, will be made continuously as long as the virus persists in the tumors. Thus, the cytokine or other immune-modulatory therapeutics encoded by OVs within the tumor milieu will be more effective and less toxic. Second, arming oncolytic viruses with immune-therapeutics is cost-effective. Immune-therapeutics such as cytokines and ICIs are very expensive, as is T-VEC, the only OV approved for clinical use. Therefore, instead of treating patients with a combination of two expensive drugs, it would be more cost-effective to use one drug that has the combined effect of both. Lastly, OVs provide a versatile platform for the expression of immuno-therapeutics. Through the use of temporally regulated viral promoters of varying transcriptional strength, it is possible to have multiple transgenes encoded from an OV with some transgenes expressed early and at high levels, and other expressed late at low levels, or vice-versa.

Although it seems logical and beneficial to arm OVs with immune-therapeutic genes instead of simply combining OVs with exogenous immune-therapeutics, the arming approach may not be applicable for all immune-therapeutics. For example, regulation of T cell function through CTLA-4 takes place at the site of T cell priming, i.e., in secondary lymphoid organs [139]. Therefore, systemic delivery of CTLA-4 inhibitors will be more effective than intratumoral delivery since systemic delivery allows more of the therapeutics to reach secondary lymphoid organs. Indeed, a study by Engeland et al. using anti-CTLA-4-armed OV found no benefit of anti-CTLA-4 expressed from virus in the tumors, whereas systemically delivered anti-CTLA-4 was able to enhance the antitumor efficacy of the parental virus. Also, a large number of preclinical studies have been published showing the enhanced efficacy of oncolytic viruses armed with PD/PD-L1 inhibitors. However, somewhat surprisingly, not one of them has entered clinical testing, whereas a plethora of studies are currently in clinical trial, testing combinations of oncolytic viruses with systemic delivery of PD-1/PD-L1 inhibitors. This again suggests that not all the immune-therapeutics may be used to arm OVs.

The efficacy of OV against solid tumors is often limited by their inability to spread within tumors due to presence of dense stroma. Several studies have tried to address this issue by arming oncolytic viruses with stromal-degrading enzymes including collagenase, hyaluronidase and decorin. While these armed viruses have shown promising results in preclinical models, it remains to be seen if such a strategy will succeed in clinical settings. Other strategies in which arming of oncolytic viruses could be taken advantage of includes the delivery of de novo targets for CAR-T cells which could make it possible to harness the benefits of CAR-T cells in the treatment of solid tumors. Furthermore, oncolytic viruses could also be armed with tumor antigens which would work as in situ vaccinations and increase the overall efficacy of the OVs. In short, the sky is the limit for what OVs can be armed with to achieve better treatments of cancer.

Future studies should focus on the clinical aspects of OVs, such as the dosing and route of administration. For example, preclinical studies have used many different dosing strategies, most of which have resulted in antitumor benefits to some extent. Given the cost associated with clinical trials, it may not be feasible to test all dosing strategies in clinical settings. Therefore, preclinical studies should reach a consensus on the dosing strategy that is most likely to result in optimal therapeutic efficacy in clinical studies. Furthermore, most preclinical studies have used the intratumoral (i.t.) route for administration; however, this may not always be feasible, especially in cases of inaccessible tumors. One reason for intratumoral delivery is to avoid virus neutralization by circulating antibodies, especially if multiple dosing is to be used. One strategy to avoid virus neutralization would be to use antigenically different strains of the virus for each dosing. In summary, more preclinical studies are needed to determine the best dosing strategy, improve virus delivery and enhance immunogenicity.

## Figures and Tables

**Table 1:** Comparison of oncolytic virus vectors.

	Adenovirus	Herpes Simplex Virus	Vaccinia Virus	Reovirus
**Genome**	dsDNA	dsDNA	dsDNA	Segmented dsRNA
**Capsid**	Icosahedral	Icosahedral	Complex	icosahedral
**Virion diameter**	70–90 nm	150–200 nm	170–200 nm	60–100 nm
**Genome size **	36–40 kb	150–200 kb	130–280 kb	0.2–3 kb
**Replication site **	Nucleus	Nucleus	Cytoplasm	Cytoplasm
**Life cycle **	24 h	12 h	8 h	18 h
**Immunogenicity**	High	Moderate	High	Low
**Transgene expression **	Transient	Potentially long-term	Transient	Transgene expression is not common
**Ease of manipulation**	Easy	Difficult	Easy	Very difficult
**Cloning capacity **	8 kb	>30 kb	25 kb	n/a
**Easily achievable titers **	10^12^ PFU/mL	10^10^ PFU/mL	10^9^ PFU/mL	10^9^ PFU/mL
**Maximum Dose used in human **	6 × 10^12^ VP [31]	4 × 10^8^ PFU [32]	3 × 10^9^ PFU [26]	3 × 10^10^ TCID_50_ [33]
**Virulence of WT virus**	Yes	Mild	Mild	No

dsDNA, Double stranded DNA; dsRNA, Double stranded RNA; kb, kilobase; PFU, plaque forming unit; VP, virus particle; TCID_50_, median tissue culture infectious dose.

**Table 2:** Armed OVs currently in clinical trials (source: clinicaltrials.gov; accessed May 2020).

Virus	Transgene	Function of Transgene	Combination	Tumor type	Phase	References
TG6002 (Vaccinia virus)	FCU1	Conversion of 5-FC to 5-FU	5-FC	Glioblastoma	Phase 1 and 2	NCT03294486
Pexa-Vec (Vaccinia virus)	GM-CSF	Activation of APC	αPD-L1 αCTLA-4	Colorectal cancer	Phase 1 and 2	NCT03206073
RP1 (HSV-1)	GM-CSF GALV-GP	Activation of APC; fusion of cells	None	Cutaneous squamous cell carcinoma	Phase 1b	NCT04349436
OH2 (HSV-2)	GM-CSF	Activation of APC	αPD-1	Gastrointestinal and other solid tumors	Phase 1	NCT03866525
T-Vec (HSV-1)	GM-CSF	Activation of APC	αPD-L1 αCTLA-4	Breast cancer	Phase 1	NCT04185311
TILT123 (Adenovirus)	TNFα and IL-2	Activation of T cells	Tumor infiltrating lymphocytes	Melanoma	Phase 1	NCT04217473
TBio-6517 (Vaccinia virus)	FLT3 ligand, IL-12 and αCTLA-4	Immune activation	αPD-1	Solid tumors, TNBC, microsatellite stable colorectal cancer	Phase 1 and 2	NCT04301011
MG1-MAGEA3 (Maraba virus)	MAGEA3	Tumor antigen for melanoma	αPD-1 Cyclophosphamide Ad-MAGEA3	Metastatic melanoma, Squamous cell skin carcinoma	Phase 1b	NCT03773744
GL-ONC1 (Vaccinia virus)	Luc-GFP fusion, β-galactosidase, β-glucuronidase	Imaging	Chemotherapy or bevacizumab	Ovarian cancer, peritoneal carcinomatosis and fallopian tube cancer	Phase 1 and 2	NCT02759588
M032 (HSV-1)	IL-12	Immune-stimulation and anti-angiogenesis	None	Glioblastoma Astrocytoma Gliosarcoma	Phase 1	NCT02062827
C134 (HSV-1)	IRS1	PKR evasion	None	Glioblastoma Astrocytoma Gliosarcoma	Phase 1	NCT03657576
TMV-018 (Measles virus)	Cytosine deaminase	Conversion of 5-FC to 5-FU	5-FC	Gastrointestinal cancer	Phase 1 and 2	NCT04195373
LOAd703 (Adenovirus)	CD40L, 41BBL	Immune stimulation	None	PDAC, ovarian cancer, biliary carcinoma and colorectal cancer	Phase 1 and 2	NCT03225989
MV-NIS (Measles virus)	NIS	Imaging	Mesenchymal stem cells transplantation	Solid tumors	Phase 1 and 2	NCT02068794
ONCR-177 (HSV-1)	IL-12, CCL4, FLT3L, αPD-L1, αCTLA-4	Immune stimulation	αPD-1	Solid tumors	Phase 1	NCT04348916
NG-350A (Adenovirus)	Anti-CD40	Immune stimulation	None	Epithelial tumors	Phase 1	NCT03852511
NG-641 (Adenovirus)	BiTE (FAP-TAc), CXCL9, CXCL10, IFNα	Killing cancer associated fibroblast; Immune stimulation	None	Epithelial tumors	Phase 1	NCT04053283

FCU1, cytosine deaminase and uracil phosphoribosyltransferase; 5-FC, 5-fluorocytosine; 5-FU, 5-fluorouracil; GM-CSF, granulocyte macrophage colony stimulating factor; GALV-GP, gibbon-ape leukemia virus glycoprotein; APC, antigen presenting cell, HSV, herpes simplex virus; TNFα, tumor necrosis factor α; IL-2, interleukin-2; IL-12, interleukin-12; FLT3, fms-like tyrosine kinase; PD-1, programmed death-1; PD-L1, programmed death ligand-1; CTLA-4, cytotoxic T lymphocyte antigen-4; MAGEA3, melanoma antigen gene A3; Luc-GFP, fusion of luciferase and GFP genes; NIS, sodium-iodide symporter; BiTE, bispecific T cell engager; FAP-Tac, fibroblast activation protein-T cell activator; IFNα, interferon α; PKR, protein kinase R.
